# Single Incision Mini-Sling Versus Mid-Urethral Sling (Transobturator/Retropubic) in Females With Stress Urinary Incontinence: A Systematic Review and Meta-Analysis

**DOI:** 10.7759/cureus.37773

**Published:** 2023-04-18

**Authors:** Tirath Patel, Fnu Sugandh, Shuaita Bai, Giustino Varrassi, Anjuli Devi, Mahima Khatri, Satesh Kumar, Deepak Dembra, Samiullah Dahri

**Affiliations:** 1 Surgery, American University of Antigua, St John, ATG; 2 Medicine, Ghulam Muhammad Mahar Medical College, Sukkur, PAK; 3 Medicine, Civil Hospital Karachi, Sukkur, PAK; 4 Medicine and Surgery, Peoples University of Medical & Health Science, Nawabshah, PAK; 5 Pain Medicine, Paolo Procacci Foundation, Rome, ITA; 6 Medicine and Surgery, Ziauddin University, Karachi, PAK; 7 Medicine and Surgery, Dow University of Health Sciences, Karachi, Karachi, PAK; 8 Medicine and Surgery, Shaheed Mohtarma Benazir Bhutto Medical College, Karachi, PAK; 9 Surgery, Ghulam Muhammad Mahar Medical College, Sukkur, PAK; 10 Medicine, Dow University of Health Sciences, Karachi, PAK

**Keywords:** sims, single incision mini-sling, stress urinary incontinence, mid-urethral sling, sui

## Abstract

Stress urine incontinence (SUI) is most common in middle-aged women and the second most common in those over 75. SUI causes significant discomfort and suffering for patients and has a considerable financial impact on the healthcare system. Conservative approaches are recommended as the first step in treatment. However, surgery is often necessary to improve a patient's quality of life due to the high failure rate of conservative treatments. A thorough literature review of studies published before March 2023 was conducted on the safety and effectiveness of single-incision mini slings (SIMS) and standard mid-urethral slings (MUS). PubMed, Embase, Cochrane Library, and Elsevier's ScienceDirect were used to retrieve the studies. Two reviewers independently searched and evaluated the data based on inclusion and exclusion criteria. Review Manager 5.4 software was used for meta-analysis. Included were seventeen studies involving 3,503 female SUI patients without intrinsic sphincter deficiency (ISD) or mixed urinary incontinence. According to the results of our meta-analysis, the clinical efficacy of SIMS is comparable to that of MUS in terms of objective cure rate (RR: 0.99; 95% CI: 0.95 to 1.03, p: 0.66, I2: 29%). In contrast, it increases the post-procedure International Consultation on Incontinence Questionnaire (ICIQ) score (WMD: 0.08; 95% CI: -0.08 to 0.08). CI: -0.02 to 0.18, p: 0.11, I2: 55%) and improves the PGI-I score to a greater extent (RR: 1.04; 95% CI: 0.96 to 1.08, p: 0.36, I2: 76%). In contrast, there is no difference between the two groups regarding patient satisfaction (RR: 0.96; 95% CI: 0.92 to 1.01, p: 0.16, I2: 0%) and Sandvik score reduction (RR: 0.98; 95% CI: 0.94 to 1.02, p: 0.35, I2: 0%). In conclusion, single-incision mid-urethral slings (SIMS) are as effective as mid-urethral slings (MUS) for treating pure stress urinary incontinence (SUI) without intrinsic sphincter deficiency (ISD), with a shorter operation time. However, the SIMS procedure has a higher incidence of dyspareunia. At the same time, bladder perforation, mesh-related complications, pelvic/groin pain, urinary tract infection (UTI), worsening urgency, dysuria, and pain score are less likely to occur with SIMS. Only the decrease in pelvic/groin pain was statistically significant.

## Introduction and background

Urinary incontinence (UI) is a prevalent illness affecting 30-50% of women throughout their lives [1]. Individuals with UI have a much lower quality of life. The incidence of stress urinary incontinence (SUI) is the highest in women under 75 and the second highest in women over 75. Prevalence estimates vary between 7 and 42 percent [2]. In addition to causing the patient much suffering and discomfort, SUI has a significant financial impact on the healthcare system. The first line of defense ought to be conservative approaches. However, since treatment failure is not unusual, surgical intervention is frequently the preferred course of action to enhance patients' quality of life [3]. For the past two decades, the mid-urethral sling has been routinely used to treat SUI successfully in women. However, retropubic and transobturator mid-urethral slings (TOT) are associated with severe adverse effects, including bladder rupture, damage to blood vessels, sellotape erosion, and pelvic or hip pain [4].

The development of single-incision mid-urethral slings (SIMS) aimed to reduce complications by shortening the insertion trajectory. Additionally, SIMS offer potential benefits such as a shorter polypropylene tape, insertion through a single vaginal incision, and the ability to perform the procedure under local anesthesia [5]. The MiniArc, a type of single-incision sling, has a self-anchoring mechanism to the pelvic sidewalls, eliminating the need for trocar passage through the obturator foramen or external skin incisions. This design could decrease postoperative pain and shorten recovery [6]. To decrease procedure-related discomfort without compromising the benefits, single-incision mini-slings (SIMS) have been created. SIMS, similar to transobturator slings, penetrate the obturator internus muscle and the foramen obturator, but they do not perforate the adductor muscles. As a result, patients may experience less pain during the postoperative period [7]. However, the current evidence regarding the efficacy and safety of single-incision mini-sling (SIMS) is still controversial [8].

Limited research has been conducted on the effectiveness and safety of single-incision mid-urethral slings (SIMS) compared to traditional mid-urethral slings. Existing studies have provided conflicting results, with only a few randomized controlled trials and observational studies available. Therefore, we conducted a systematic review and meta-analysis to determine which surgical procedure is superior for treating stress urinary incontinence. As far as we know, this is the first recently updated meta-analysis to compare SIMS versus mid-urethral slings for treating this condition.

## Review

Methods 

The Preferred Reporting Items for Systematic Review and Meta-analysis (PRISMA) guidelines [9] were followed in drafting this meta-analysis. 

Search Strategy

Electronic searches without language constraints were conducted on PubMed, Embase, the Cochrane Library, and Elsevier's ScienceDirect databases for clinical research (updated in March 2023). For literature retrieval, simple keyword and medical subject heading (MeSH) term combinations (such as "single-incision mini-sling," "Contasure-Needleless," "needleless," "trans obturator slings," "TVT-O," "TOT," etc.) were utilized. In addition, thorough searches of each pertinent review's references and citation lists were conducted. The population, intervention, comparison, and outcome (PICO) methodology was followed. Women with stress urinary incontinence represented the population of interest (SUI). Three researchers (T.P, F.S, and F.S.B) assessed the titles and abstracts of possibly eligible studies separately. 

Inclusion And Exclusion Criteria

The included studies met the following criteria: The studies must be either randomized controlled trials (RCTs) or observational studies (cohort or case-control). The researchers compared the efficacy and safety of single-incision mini-slings (SIMS) to traditional synthetic mid-urethral slings (MUS). Participants were SUI-positive females, and there was no statistically significant difference in the fundamental characteristics of the participants. The outcomes included the cure rate, surgery-related data, and postoperative sequelae.

The following criteria were used to exclude studies: Studies or articles that do not provide sufficient data for meta-analysis, studies that are not original research such as conference abstracts, case reports, case series studies, editorials, or review articles, studies with a follow-up time of less than one year, and studies that focus on patients diagnosed with intrinsic urethral sphincter deficiency (ISD). The exclusion of studies with ISD is necessary as it is a distinct condition from stress urinary incontinence, which is the focus of this meta-analysis.

Data Extraction And Definitions

The literature selection was completed according to the inclusion and exclusion criteria. Two reviewers (T.P and F.S) independently extracted data and appraised quality and content. The following items were extracted from each available study: first author, year of publication, country, study design, intervention, sample size, follow-up data, baseline variables (age, gender, body mass index (BMI), parity), and relative outcome (including subjective cure rate, objective cure rate, operative time, hospitalization time, blood loss, visual analog scale) and overall complications. 

The primary outcomes were objective cure rate and subjective cure rate. The negative cough stress test analyzed the objective cure rate, whereas the subjective cure rate comprised of patient global impression of improvement scale (PGI-I), patient satisfaction, postoperative Sandvik score, and international consultation on incontinence score (ICIQ). The secondary outcomes included adverse events related to the two procedures, the need for revision surgery/re-surgery, operative time, and length of hospital stay. 

Cough stress test: The patient was supine/lithotomy and had 200-400 mL of fluid in the bladder. The examiner directly visualized the urethral meatus for the presence of leaking after she coughed 1-4 times. Fluid leakage from the urethral meatus that occurs concurrently with/simultaneously with the cough(s) is considered a positive test. The subjective cure rate was defined as "very much better" or "much better" based on the PGI-I, postoperative ICIQ-SF cut-off score (of 6/21) that is likely to be associated with a patient-reported successful outcome on the PGI-I following surgical therapy. Sandvik score: The score range is 0 to 8, with 0 being the lowest and 8 being the highest (or 12 for the fourth level). The greater the score, the more severe the incontinence. 

Quality Of Included Studies

Quality assessment of all the included RCTs and observational studies was done by using the Cochrane risk of bias tool [10] and Newcastle-Ottawa scale [11], respectively. 

Statistical Analysis

Only comparative studies were statistically analyzed with Review Manager 5.4.1. (The Nordic Cochrane Centre, The Cochrane Collaboration, 2014, Denmark). This meta-analysis calculates a pooled effect of relative risks (RRs) for dichotomous outcomes and weighted mean differences (WMDs) for continuous outcomes using the generic-inverse variance with a random-effects model. The findings of the pooled analysis were illustrated using forest plots. Funnel plots of primary outcomes were produced for each primary outcome to evaluate publication bias. Using Higgin's I2 test [12], levels of heterogeneity of low (25%), moderate (25-75%), and high (> 75%) were determined. A sensitivity analysis was performed to assess the influence of the individual studies on the overall results by omitting one study at a time when substantial heterogeneity (I2 >75%) was present. A univariate linear meta-regression was performed to determine the relationship between outcomes such as a negative cough stress test and a Sandvik score lower than the pre-operative score and baseline variables such as age, BMI, and parity. If the p-value was less than 0.05, all analyses were declared significant. 

Since the data were acquired and analyzed from previous clinical studies for which the researchers had already obtained informed consent, no approval from an ethical committee was necessary for this investigation.

Results

Characteristics OF The Eligible Studies 

**Figure 1:**
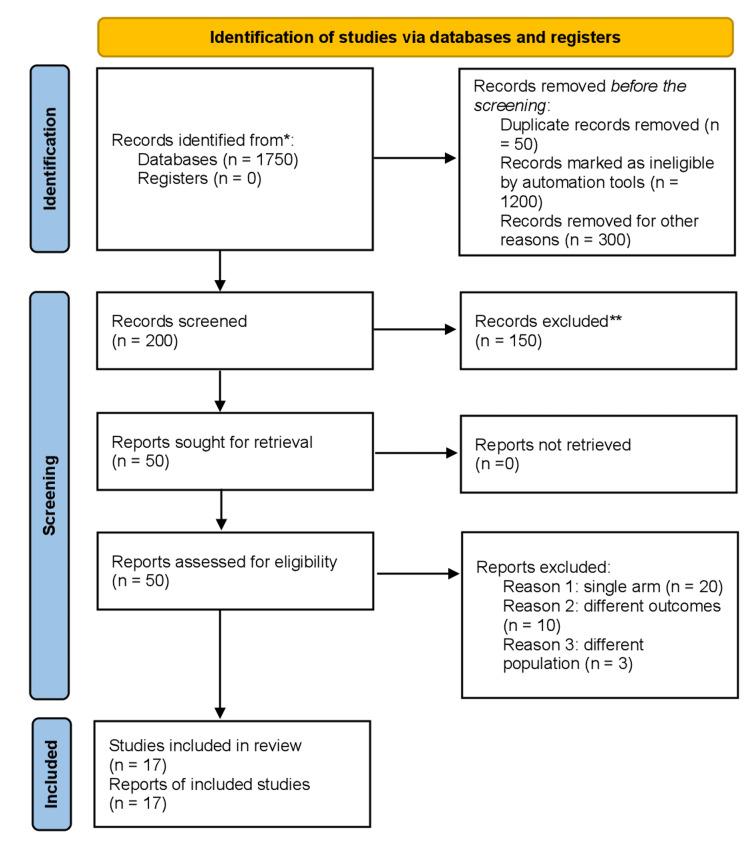
Prisma flow chart Preferred Reporting Items for Systematic Review and Meta-analysis flow chart

The literature review initially yielded 1750 articles. After removing duplicates and screening studies based on their titles and abstracts, seventeen [13-29] studies were found, including retrospective and prospective ones. Comparative studies comprised the entirety of those included in this meta-analysis. The PRISMA diagram illustrates a comprehensive search strategy, as shown in Figure 1. This collection of articles spans the years 2011 through 2023. Six of the seventeen articles were observational cohort studies [20,22-24,26,27], and the other eleven were controlled trials [13-19,21,25,28,29]. Of the eleven controlled trials, nine were randomized controlled trials (RCTs), one was a controlled clinical trial (CCT) [18], and one was a quasi-randomized trial [15]. Seven included studies [13-18, 21] were single-center, and the remaining were multicenter. All studies included a transobturator mid-urethral sling in the control group, except for two studies [25,26] that included a public mid-urethral sling along with the transobturator sling in the control group. The mean follow-up duration was 33 months.

Baseline Characteristics Of The Patients 

The overall number of participants was 3503 (1773 in the SIMS group and 1730 in the MUS group), with mean ages ranging from 44.1 ± 7.0 to 62.5 ± 10.4. All the patients included in the study were females with SUI. Most patients included were overweight, with an average parity of 3 ± 1.1. Tables 1-2 summarize the patients' features.

**Table 1:** Baseline demographics of the included participants SD: Standard deviation, SIMS: Single-incision mini-slings, MUS: Mid-urethral slings, NA: Not Available, BMI: Body mass index

Study	Study design	Country	Follow-up (months)	Total No. of patients	No. of patients		Mid-urethral sling type		Age (mean ±SD)		BMI (Kg/m2) (Mean ±SD)		Parity No. (%)	
					SIMS	MUS	Retropubic	Transobturator	SIMS	MUS	SIMS	MUS	SIMS	MUS
Dogan (2018) [13]	single-center prospective RCT	Turkey	24	178	89	89	N/A	89	49.03 ± 9.18	51.92 ± 6.98	27.94 ± 5.03	26.61 ± 3.87	3 (0–9)	3 (1–6)
Fernandez (2016) [14]	single-center prospective RCT	Spain	12	187	89	98	N/A	98	57.6 ± 11.03	57.8 ± 57.83	28.7 ± 4.97	28.1 ± 4.44	2 (0–6)	2 (0–8)
Franco E (2015) [15]	single-center prospective RCT	Spain	60	239	131	108	N/A	108	58.9 ± 12.7	58.5 ± 11.75	28.05 ± 5.5	28.8 ± 5.6	2.48 (0–7)	2.64 (0–9)
Gaber (2016) [16]	single-center prospective RCT	United Kingdom	12	140	70	70	N/A	70	44.1 ± 7.0	44.3 ± 8.5	26.5 ± 2.5	25.7 ± 2.4	3 (3–4)	3 (3–4)
Lv (2017) [17]	single-center prospective RCT	China	12	164	78	86	N/A	86	52.3 ± 10.02	52.43 ± 10.86	26.04± 3.46	25.85± 3.71	N/A	N/A
Tardiu (2011) [18]	single-center prospective CCT	Spain	12	132	72	60	N/A	60	59.9 ± 9.07	60.6 ± 8.34	29.13± 3.76	29.01± 4.19	2.53 (0–6)	2.63 (0–9)
Xu (2017) [19]	Multicenter prospective RCT	China	12	148	74	74	N/A	74	56.3 ± 8	57 ± 9	28± 3.8	28± 3.5	N/A	N/A
White (2020) [20]	observational cohort multi-center study	Multi-center	36	281	141	140	N/A	140	49.1 ± 11.6	48.9 ± 11.7	29.6± 7.3	29.7± 6.3	N/A	N/A
Maturana (2020) [21]	single-center prospective RCT	Brazil	12	105	58	47	N/A	47	55.6 ± 1.5	55.7 ± 1.8	28.4 ± 0.6	28.9 ± 0.6	4.3 (0.3)	4.6 (0.4)
Zhang (2020) [22]	observational cohort	China	36	107	51	56	N/A	56	58.8 ± 9.3	56.9 ± 11.4	N/A	N/A	N/A	N/A
Akdemir (2020) [23]	observational cohort	Turkey	12	79	39	40	N/A	40	52.4± 8.48	52.1 ± 9.57	30.36± 4.48	31.13± 5.03	3.38 (1.61)	3.35 (1.51)
B White (2021) [24]	observational cohort	United States and Australia	30	281	141	140	N/A	140	56.6 ± 10.25	56.5 ± 10.7	30 ± 6.6	29.8± 5.9	N/A	N/A
Fattah M (2022) [25]	Multicenter prospective RCT	United Kingdom	36	596	298	298	119	38	50.4 ± 11.0	50.7 ± 10.9	28.9 ± 5.5	28.7 ± 5.6	2.4 (1.1)	2.4 (1.1)
Erickson T (2020) [26]	observational cohort multi-center study	United States and Canada	36	355	184	171	85	89	56.2 ± 11.4	53.3 ± 12.3	30 ±5.8	31.8± 7.6	2 (0–7)	2 (0–7)
Sun Z (2019) [27]	observational cohort	China	120	64	33	31	N/A	31	55.8 ± 9.1	58.7 ± 6.3	23.8 ± 4.8	24.3 ± 2.6	1.5 (0.8)	1.2 (0.5)
Huser M (2023) [28]	Multicenter prospective RCT (open-label)	United States and Canada	48	168	84	84	N/A	84	61.3 ± 10.0	62.5 ± 10.4	28.7 ± 6.7	29.5 ± 6.0	2.2 (1.2)	2.3 (1.1)
Alexandridis V (2019) [29]	Multicenter prospective RCT	Multi-center	36	279	141	138	N/A	138	44.9 ± 6.8	45.9 ± 7.3	26.2 ± 4.8	26.6 ± 4.6	2.0 ± 1.0	2.0 ± 1.0

**Table 2:** Baseline demographics including previous surgical histories N/A: Not Available, SIMS: Single-incision mini-slings, MUS: Mid-urethral slings

Study	Current smoker No. (%)		Postmenopausal No. (%)		Previous pelvic surgery No. (%)		Previous prolapse surgery No. (%)		Previous hysterectomy No. (%)	
	SIMS	MUS	SIMS	MUS	SIMS	MUS	SIMS	MUS	SIMS	MUS
Dogan (2018) [13]	N/A	N/A	37 (41.5)	42 (47.1)	N/A	N/A	N/A	N/A	N/A	N/A
Fernandez (2016) [14]	16 (18)	6 (6.1)	69 (77.5)	61 (62.2)	42 (47.2)	41 (41.8)	39 (43.8)	44 (44.8)	23 (25.8)	22 (22.4)
Franco E (2015) [15]	11 (8.3)	14 (13)	108 (82.4)	77 (71.2)	N/A	N/A	79 (60.3)	75 (69.4)	40 (30.5)	42 (38.8)
Gaber (2016) [16]	N/A	N/A	23 (32.9)	23 (32.9)	N/A	N/A	N/A	N/A	N/A	N/A
Lv (2017) [17]	N/A	N/A	N/A	N/A	N/A	N/A	N/A	N/A	N/A	N/A
Tardiu (2011) [18]	5 (6.9)	11 (18.3)	N/A	N/A	N/A	N/A	N/A	N/A	N/A	N/A
Xu (2017) [19]	N/A	N/A	24 (32.4)	25 (33.7)	N/A	N/A	N/A	N/A	N/A	N/A
White (2020) [20]	13 (9.2)	22 (15.7)	69 (48.9)	60 (42.9)	93 (66.9)	82 (59)	50 (35.9)	39 (28)	40 (28.4)	34 (24.3)
Maturana (2020) [21]	N/A	N/A	N/A	N/A	N/A	N/A	N/A	N/A	N/A	N/A
Zhang (2020) [22]	N/A	N/A	37 (63.8)	32 (68)	N/A	N/A	N/A	N/A	N/A	N/A
Akdemir (2020) [23]	12 (30.8)	7 (17.5)	18 (46.2)	21 (52.5)	N/A	N/A	34 (87.1)	28 (70)	17 (43.5)	19 (47.5)
B White (2021) [24]	13 (9.2)	20 (14.2)	69 (48.9)	54 (38.5)	93 (65.9)	82 (58.5)	55 (39)	53 (37.8)	40 (28.3)	29 (20.7)
Fattah M (2022) [25]	52 (17.4)	43 (14.4)	N/A	N/A	N/A	N/A	N/A	N/A	N/A	N/A
Erickson T (2020) [26]	17 (9.2)	33 (19.3)	134 (72.8)	101 (59.1)	129 (70.1)	116 (67.8)	6 (3.2)	6 (3.5)	74 (40.2)	69 (40.4)
Sun Z (2019) [27]	N/A	N/A	26 (78.8)	29 (93.5)	N/A	N/A	N/A	N/A	N/A	N/A
Huser M (2023) [28]	N/A	N/A	N/A	N/A	N/A	N/A	N/A	N/A	N/A	N/A
Alexandridis V (2019) [29]	25 (18.0)	21 (15.4)	N/A	N/A	N/A	N/A	3 (2.1)	2 (1.4)	15 (10.7)	15 (10.9)

Quality Assessment And Publication Bias

**Figure 2:**
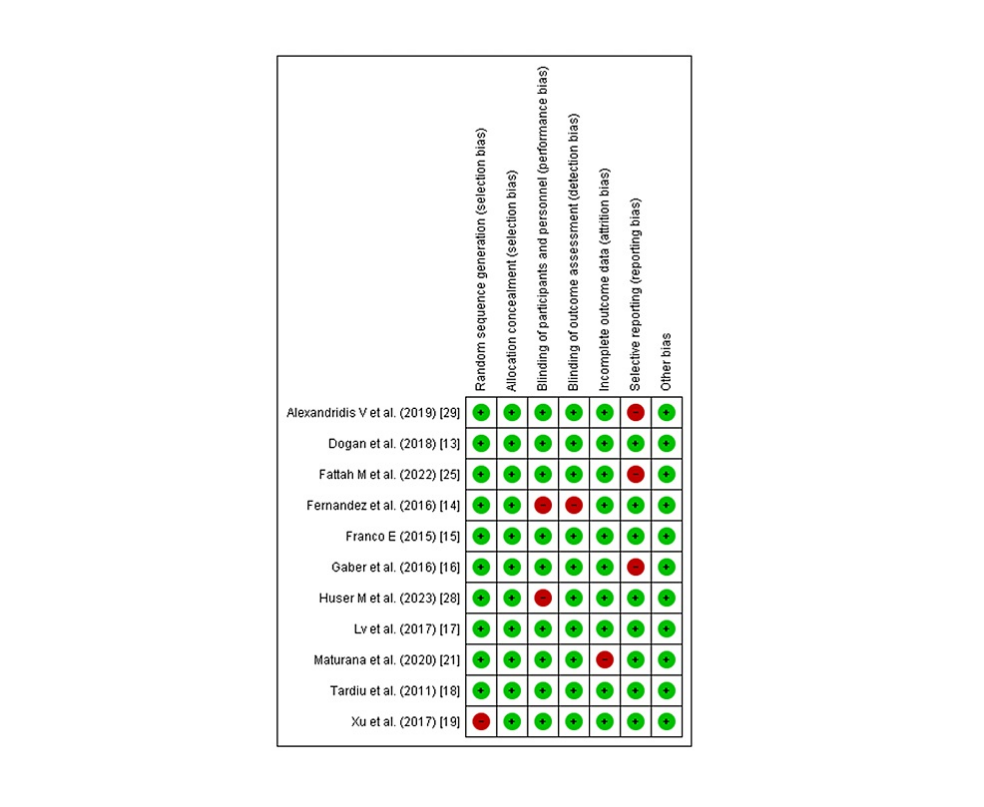
Quality assessment of included Randomized controlled trials (RCTs) Source: References: [13-19,21,25,28,29]

**Figure 3:**
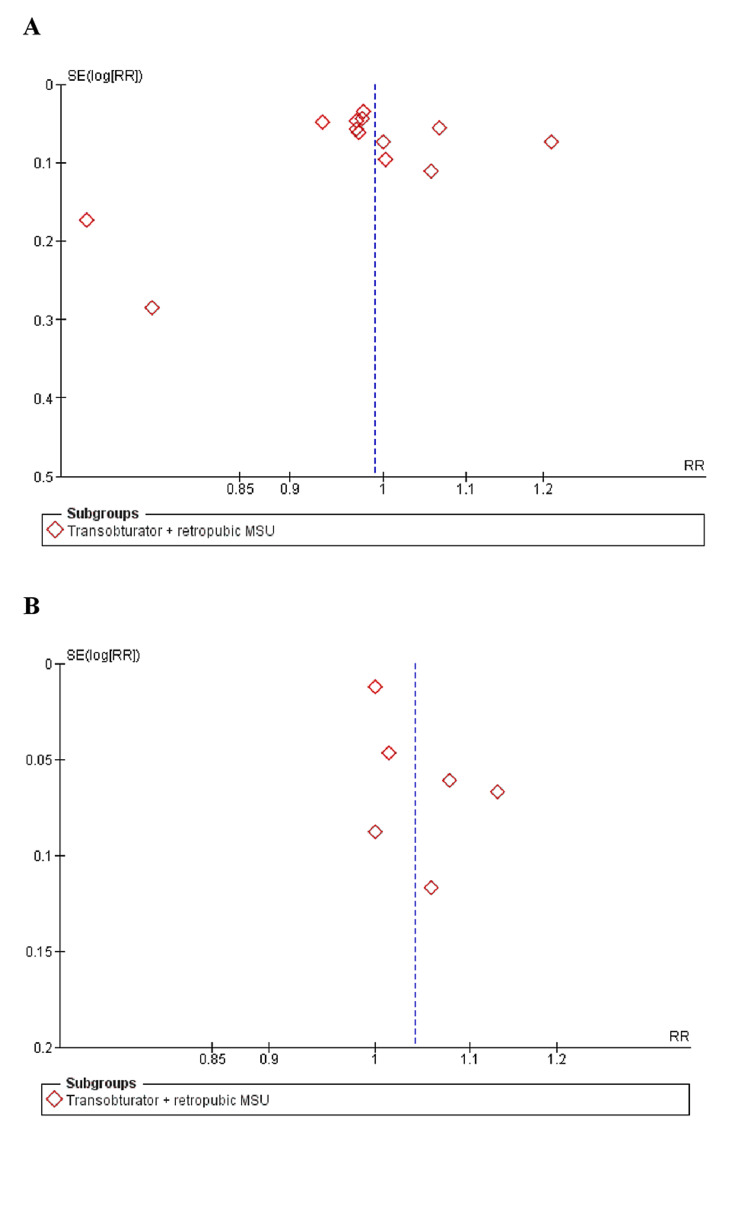
Funnel plots of primary outcomes (A) Negative cough stress test (B) PGI-I (better or very much better) SE: Standard error, RR: Relative risk

**Figure 4:**
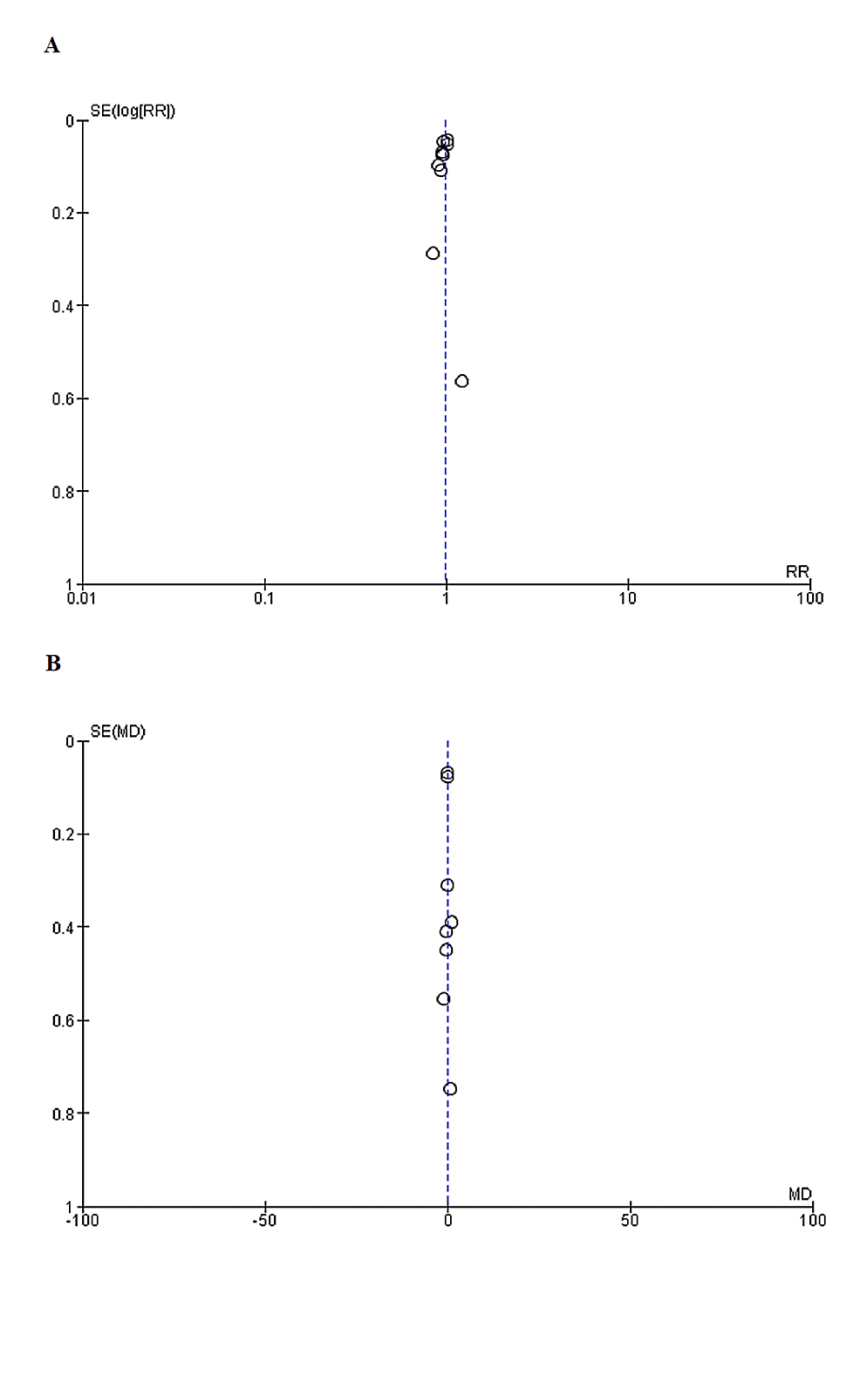
Funnel plots: (A) Sandvik score > than the preoperative score (B) Post procedure ICIQ score SE: Standard error, WMD: Weighted mean difference, RR: Relative risk

**Figure 5:**
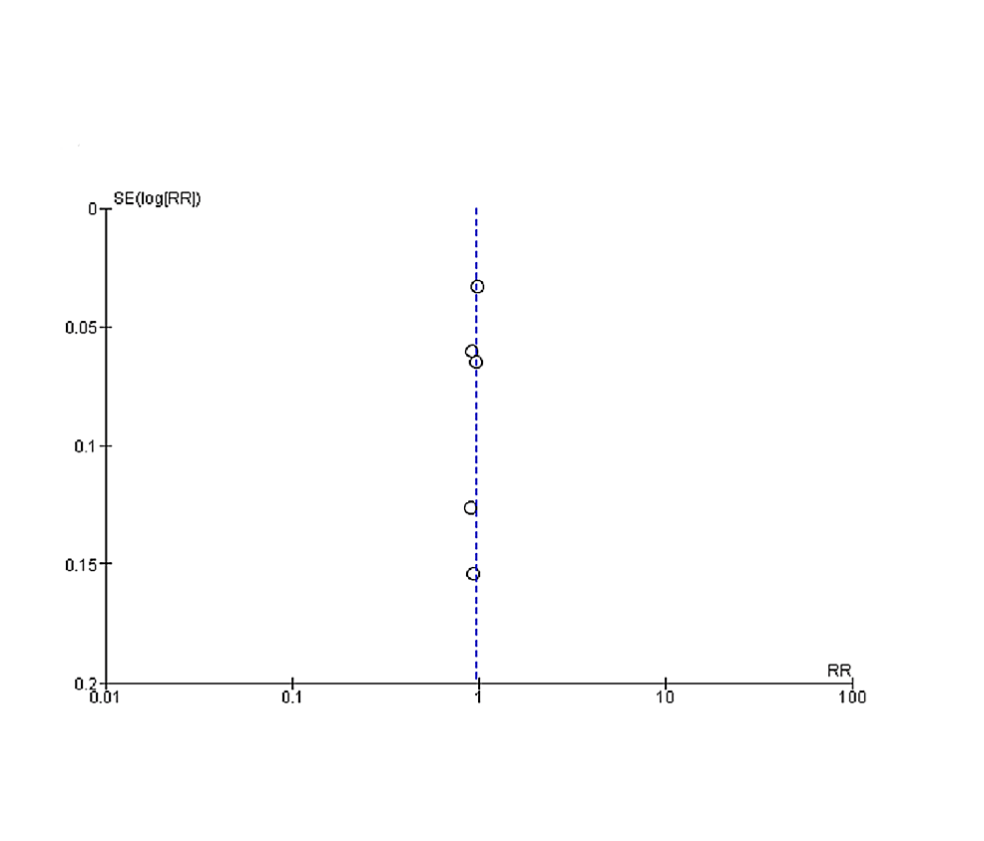
Funnel plots representing Patient Satisfaction SE: Standard error, RR: Relative risk

As determined by the New Castle-Ottawa scale, observational studies have a low probability of bias, a technique for assessing study quality, as shown in Table 3. We uncovered moderate to high-quality trials using the Cochrane approach for assessing RCTs, as shown in Figure 2. As seen by funnel plots of primary outcomes, the results were unaffected by publication bias, as shown in Figures 3, 4, 5.

**Table 3:** New Castle Ottawa scale to assess Publication bias in Observational studies The Newcastle-Ottawa Scale quality instrument is scored by awarding a point for each answer that is marked with an asterisk below. Possible total points are 4 points for Selection, 2 points for Comparability, and 3 points for Outcomes. Good quality: 3 or 4 stars in the selection domain AND 1 or 2 stars in the comparability domain AND 2 or 3 stars in the outcome/exposure domain Fair quality: 2 stars in the selection domain AND 1 or 2 stars in the comparability domain AND 2 or 3 stars in outcome/exposure domain Poor quality: 0 or 1 star in selection domain OR 0 stars in comparability domain OR 0 or 1 stars in outcome/exposure domain.

Study	Selection				Comparability	Outcomes			Total
	Representativeness of the Exposed Cohort	Selection of the Non-Exposed Cohort	Ascertainment of Exposure	Demonstration That Outcome of Interest Was Not Present at Start of Study	Comparability of Cohorts on the Basis of the Design or Analysis	Assessment of Outcome	Was Follow-Up Long Enough for Outcomes to Occur	Adequacy of Follow-Up of Cohorts	
White, et al (2020) [20]	*	*	*	*	**	*	*	*	*********
Zhang, et al (2020) [22]	*	*	*	*	*	*	*	*	********
Akdemir, et al (2020) [23]	*	*	*	*	**	*	*	*	*********
B White, et al (2021) [24]	*	*	*	*	*	*	*	*	********
Erickson T, et al (2020) [26]	*	*	*	*	**	*	*	*	*********
Sun Z, et al (2019) [27]	*	*	*	*	*	*	*	*	********

Primary Outcomes 

The objective cure rate and the subjective cure rate characterized the primary outcomes. The subjective cure rate was determined by patient satisfaction, PGI-I (better or very much better), Sandvik score < than the pre-operative score, and ICIQ score. The objective cure rate was determined primarily by analyzing patients with a negative cough stress test. 

**Figure 6:**
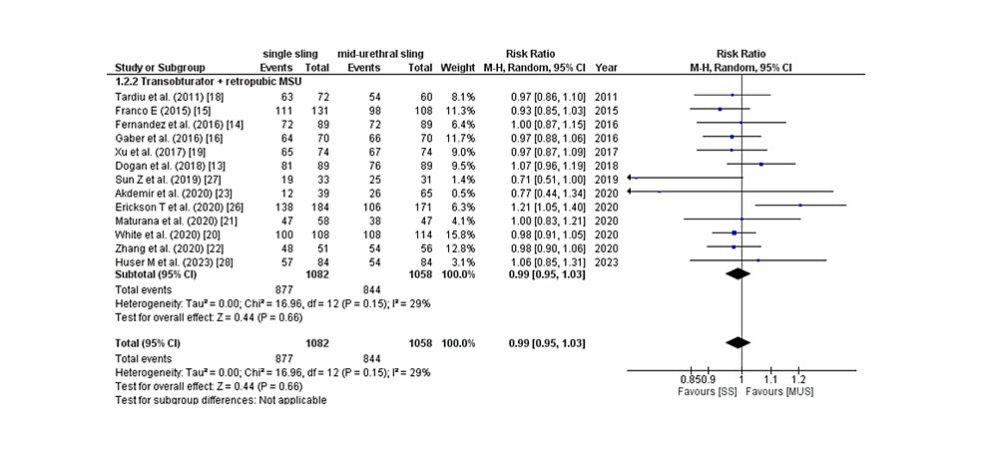
Negative cough stress test CI= confidence interval, I2= Heterogeneity Source: References: [13-16,18-23,26-28].

Objective cure rate: Thirteen of 17 studies provided the objective cure rate, which was predominantly a negative cough stress test, and the pooled analysis revealed that the rate of the negative cough stress test was comparable across the two groups (RR: 0.99; 95% CI: 0.95 to 1.03, p: 0.66, I2: 29%) as shown in Figure 6.

**Figure 7:**
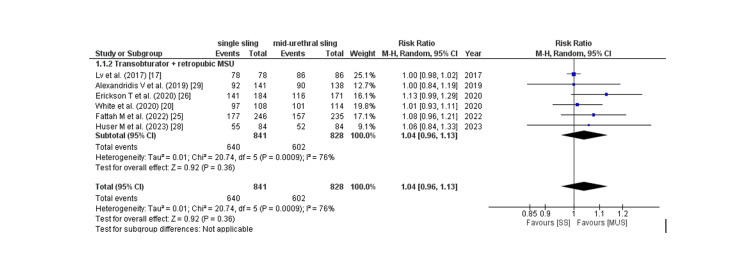
PGI-I (better or very much better) CI= confidence interval, I2= Heterogeneity Source: References: [17,20,25,26,28,29].

**Figure 8:**
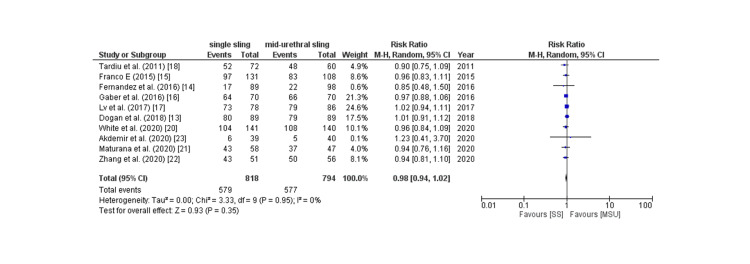
Sandvik score > than the preoperative score CI= confidence interval, I2= Heterogeneity Source: References: [13-18,20-23]

**Figure 9:**
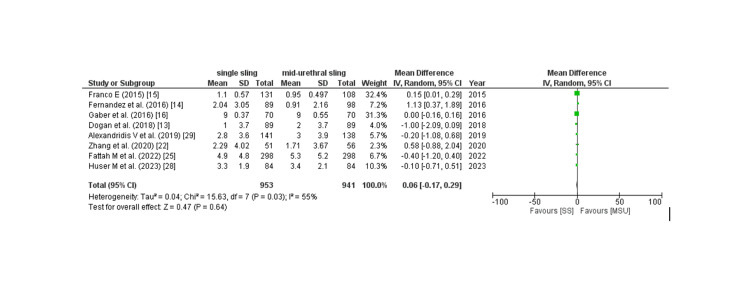
Post procedure ICIQ score WMD= weighted mean difference, CI= confidence interval Source: References: [13-16,22,25,28,29]

**Figure 10:**
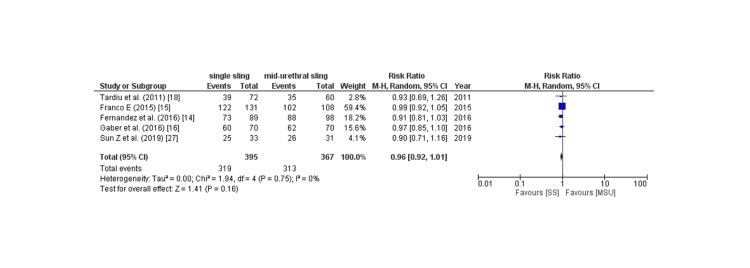
Patient Satisfaction CI= confidence interval, I2= Heterogeneity Source: References: [14-16,18,27]

Subjective cure rate: The subjective cure rate was analyzed by assessing four outcomes: PGI-I score, Sandvik score, postoperative ICIQ score, and patient satisfaction, which are described as follows: Six of 17 studies provided PGI-I score, and the pooled analysis revealed that patients in the SIMS group had a non-significantly increased rate of PGI-I score better than baseline compared to placebo (RR: 1.04; 95% CI: 0.96 to 1.08, p: 0.36, I2: 76%) as shown in Figure 7. Owing to the high degree of heterogeneity, a leave-one-out sensitivity analysis was conducted, which revealed that removing the trial by lv et al. [17] considerably reduced within-study heterogeneity (RR: 1.05; 95% CI: 0.99 to 1.12, p: 0.09, I2: 0%). 10 of 17 studies provided the Sandvik score, and the pooled analysis found no significant difference between the two groups in terms of the Sandvik score (RR: 0.98; 95% CI: 0.94 to 1.02, p: 0.35, I2: 0%) as shown in Figure 8. Eight of 17 studies provided the post-procedure ICIQ scores. The pooled analysis revealed that the SIMS technique was related to a non-significant modest rise in the post-procedure ICIQ score (WMD: 0.08; 95% CI: -0.08 to 0.08). CI: -0.02 to 0.18, p: 0.11, I2: 55%) as shown in Figure 9. Five out of the 17 studies provided the data on patient satisfaction, and the pooled analysis showed that there was no significant difference between the two groups as for patient satisfaction (RR: 0.96; 95% CI: 0.92 to 1.01, p: 0.16, I2: 0%) as shown in Figure 10.

Secondary Outcomes 

The secondary outcomes were the length of the operation, the length of the hospital stay, the necessity for revision or surgery, and adverse events. A list of secondary outcomes is included in Table 4.

**Table 4:** Secondary Outcomes UTI- urinary tract infection; VAS –visual analogue scale; RR- Relative risk; WMD- weighted mean difference; CI- confidence interval; I2- Heterogeneity.

Outcome	Effect size	95% CI	p-value	I2
Operative time	WMD: -5.46	-8.51, -2.41	0.0004	98%
Hospital stay	WMD: -0.34	-1.03, 0.35	0.33	98%
Re-surgery	RR: 0.86	0.40, 1.82	0.69	44%
Adverse events				
Urinary retention	RR: 0.96	0.56, 1.65	0.89	0%
Bladder perforation	RR: 0.37	0.12, 1.08	0.07	0%
Mesh related complications	RR: 0.75	0.41, 1.36	0.34	0%
Pelvic/ groin pain	RR: 0.54	0.30, 0.97	0.04	33%
UTI	RR: 0.63	0.36, 1.10	0.11	0%
Dyspareunia	RR: 1.76	1.03, 2.98	0.04	0%
Urgency worsening	RR: 0.83	0.53, 1.31	0.43	20%
Dysuria	RR: 0.69	0.17, 2.82	0.60	0%
Pain score > 5 on VAS	RR: 0.40	0.16, 0.99	0.05	27%

Operative time: The operative time was provided by 7 of the 17 studies, and the pooled analysis revealed that the SIMS procedure required much less time than the MUS treatment. Owing to the significant level of heterogeneity, a leave-one-out sensitivity analysis was conducted, which revealed that a single study did not influence the results of this outcome. 

Length of hospital stay: Seven of the 17 studies recorded the length of hospital stay. The pooled analysis revealed that the SIMS technique was related to a non-significantly shortened length of hospital stay. Due to the considerable heterogeneity, a leave-one-out sensitivity analysis was performed, which demonstrated that any single study did not affect the results of this outcome. 

Need for revision/re-surgery: Five of 15 studies provided the rate of revision surgery or re-surgery, and the pooled analysis revealed that the SIMS procedure was associated with a non-significantly lower risk of revision/re-surgery than the MUS approach. 

Adverse events: The pooled analysis of adverse events revealed that the SIMS procedure was linked with a significantly higher risk of dyspareunia when compared with the conventional MUS procedure. On the other hand, it was linked to a lower risk of bladder perforation, mesh-related complications, pelvic/groin pain, urinary tract infection (UTI), urgency worsening, dysuria, and a pain score < 5 on the visual analog scale (VAS). Still, only the reduction in pelvic/groin pain was statistically significant. The rate of urine retention did not differ between the two groups. 

*Univariate Meta-Regression* 

The study used univariate linear meta-regression analysis to investigate the correlation between adverse cough stress test outcomes and Sandvik scores less than the pre-operative scores and baseline variables, including age, BMI, and parity. The results indicated that the negative cough stress test rate decreased with age but increased with parity and BMI, although these results did not reach statistical significance. On the other hand, the rate of Sandvik score being less than the pre-operative score decreased with increasing BMI but increased with age and parity. Table 5 summarizes the study's findings from the univariate meta-regression analysis.

**Table 5:** Univariate meta-regression BMI- Body mass index

Outcome	Covariates	Co-efficient	p-value
Negative Cough stress test	Age	-0.0018	0.657
	Parity	-0.0221	0.5923
	BMI	0.0275	0.06
Sandvik score < than the pre-operative score	Age	0.0018	0.73
	Parity	0.059	0.32
	BMI	-0.015	0.486

Discussion 

Petros and Ulmsten introduced the tension-free vaginal tape (TVT) in 1995 as a simple and highly effective surgical method for treating female stress urinary incontinence (SUI). In recent years, there have been significant advancements in treating female SUI, and TVT has become a popular procedure due to its excellent efficacy and short learning curve. This popularity has also led to the development of various procedure modifications [30]. The introduction of the International Consultation on Incontinence Questionnaire-Urinary Incontinence Short Form (ICIQ-UI SF) and Patient Global Impression of Improvement (PGI-I) marked a significant breakthrough in SUI surgery. In addition to the nonvalidated structured questionnaires routinely used for clinical follow-up of pelvic floor dysfunction and urogynecological surgery in our unit, these validated questionnaires were also employed [31]. The incidence of stress urinary incontinence (SUI) and increased knowledge about its pathophysiology have resulted in several surgical methods for its treatment. The latest surgical advancements primarily involve synthetic slings [32]. Tension-free Vaginal Tape (TO-TVT) is the second generation of tension-free sling, succeeding the first-generation retropubic sling approach, which has evolved into the TVT and SPARC procedures. The next-generation sling via the obturator also has two procedures: outward-inward and inward-outward (TVT-O). The third generation of the sling is the Single Incision Mini-sling (SIMS), which has high cure rates similar to RP-TVT and TO-TVT but with fewer postoperative complications and improved safety. SIMS includes ten types: TVT-Scure, Miniarc, Ajust, Cure mesh, C-NDL, TFS, Ophria, MiniTap, Altis, and Solyx. However, TVT-Secur has been withdrawn from clinical practice due to poor efficacy. At the same time, Cure Mesh, MiniTap, Altis, and Solyx have no promising prospects [33]. 

This comprehensive systematic review and meta-analysis of 17 studies included 3503 participants. They aimed to analyze and compare the safety and effectiveness of two surgical techniques: single incision mini-sling (SIMS) and Mid-urethral sling. In addition, it was essential to evaluate intraoperative and postoperative problems. Regarding demographic and pre-operative factors, the groups were comparable clinically before surgery. Considering primary outcomes, the objective cure rate was almost similar in both groups. According to Bakas et al.2's research, the TVT procedure effectively treats SUI in women with pure SUI and a cystocele of no more than grade 1. The study showed that the procedure has an 83.9% objective cure rate and a 78.6% subjective cure rate at a 17-year follow-up and has a minimal risk of complications [34]. The subjective cure rate in our study was determined based on several factors, including patient satisfaction, an improvement of PGI-I scores to "better" or "very much better," an increase in Sandvik scores compared to pre-operative levels, and a decrease in ICIQ scores after the procedure. None of the factors were found to be statistically significant. The MiniArc device has demonstrated excellent performance over time, as evidenced by Lo et al.'s study showing high subjective and objective cure rates of 83% and 88% at three and five years, respectively.

Furthermore, there were no significant differences in patient-reported and objective cure rates between the currently used SIMS during midterm follow-up [35]. However, there are limited studies available on the long-term outcomes of SIS. Sun et al. conducted a study comparing TVT-O patients and SISs (TVT Secur) ten years after implantation. They found that transobturator tape is superior in objective cure and subjective satisfaction and has less decline over ten years. One patient required reoperation for failure and received a retropubic mid-urethral sling. The mean PGI-I scores and ICIQ-SF were 1.5 (± 1.0) and 3.2 (± 4.8), respectively, while patients' satisfaction was scored at 8.6 (± 2.6) out of 10 [35]. No patients underwent tape cut for persistent positive postvoid residual volume or mesh removal, and no long-term complications occurred. The study compared long-term cure rates and functional outcomes with short-term outcomes for the same patients available through their previous database [35].

The secondary outcomes of the study comprised the duration of the operation, hospitalization period, requirement for revision or surgery, and any unfavorable incidents. The SIMS procedure had a significantly shorter operative time than the MUS treatment, and the other factors were discovered to be almost insignificant. In contrast, adverse events associated with the SIMS procedure were significantly linked with a higher risk of dyspareunia than the conventional MUS procedure. The combination of design features in the Altis procedure is distinctive. While direct comparisons with other SIMS have yet to be conducted, it is crucial to comprehend the theoretical principles of each design. Given the many surgical options available to surgeons, the advantages of SIMS should be considered from a patient's viewpoint. SIMS generally has shorter operation times, reduced postoperative pain, and faster recovery. Furthermore, apart from TVT-Secur, the effectiveness of SIMS is not inferior to conventional mid-urethral slings [36].

Our study had several advantages (1). A total of seventeen studies, comprising both randomized controlled trials and observational cohorts, reinforced the meta-analysis we conducted. This significantly impacted the overall sample size and increased the study's power. (2) To examine how various studies influenced the overall estimate, a sensitivity analysis was conducted to assess PGI-I (better or very much better), operative time, and length of hospital stay to check for high heterogeneity. (3) To assess publication biases, a range of tests and plots, such as funnel plots, were employed, and none were significant. (4) Furthermore, the Newcastle-Ottawa Scale was used to identify any publication bias in the additional observational study included in our meta-analysis. (5) This study used a univariate linear meta-regression analysis to examine the relationship between adverse cough stress test outcomes and Sandvik scores and baseline variables, including age, BMI, and parity. While the negative cough stress test rate decreased with age, it increased with parity and BMI. However, these findings were not statistically significant. Conversely, the rate of Sandvik scores being less than pre-operative scores decreased with increasing BMI. In contrast, it increased with age and parity.

While our study produced significant statistical data, it is vital to recognize its limitations. (1) Firstly, most studies' follow-up durations varied significantly, with some indicating longer durations. In evaluating any surgical procedures, longitudinal follow-up studies are preferred; thus, a longer follow-up period would have been preferable. (2) The observed clinical heterogeneity may have been caused by variations in study designs, interventions, and patient factors (such as BMI, age, sample size, ethnicity, and trial characteristics). (3) The lack of a clear and detailed description of blinding procedures in the included RCTs may have resulted in conclusion bias. The occurrence of potential biases resulting from CCTs is inevitable. (4) Furthermore, assessing technical equipment and surgical proficiency is imperative for evaluating the efficacy of the two approaches. Yet, it was not feasible to appraise them in the current review. (5) An additional constraint of this study is the inclusion of unselected populations in some of the studies (patients with pelvic organ prolapse and varying degrees of stress urinary incontinence) and the possibility that some patients were duplicated across two studies.

## Conclusions

To summarize, single-incision mid-urethral slings (SIMS) exhibit comparable short-term efficacy to mid-urethral slings (MUS) in treating patients with pure stress urinary incontinence (SUI) and no signs of intrinsic sphincter deficiency (ISD). Compared to mid-urethral slings, the SIMS procedure demonstrated a shorter operative time and a significantly higher incidence of dyspareunia. Conversely, it was associated with a decreased risk of bladder perforation, mesh-related complications, pelvic/groin pain, urinary tract infection (UTI), worsening urgency, dysuria, and a pain score of less than five on the visual analog scale (VAS). However, only the decrease in pelvic/groin pain showed statistical significance. Consideration of the identified limitations is essential when interpreting the results. Large, well-designed prospective randomized controlled trials with extensive follow-up are necessary to confirm the long-term efficacy and safety of the intervention.
